# Supplementary material for: Acute kidney injury and acute kidney recovery following Transcatheter Aortic Valve Replacement
Source: PLoS One. 2021 Aug 10;16(8):e0255806. doi: 10.1371/journal.pone.0255806 (PMC8354447; doi:10.1371/journal.pone.0255806)
Supplement: S1 Table — (DOCX) [file pone.0255806.s001.docx]

**S1 table (Supplementary Table 1): AKI. AKR and Unchanged renal function according to the time of procedure**

| **Year** | **Global Cohort** | **Unchanged** | **AKI** | **AKR** | **p value** |
| --- | --- | --- | --- | --- | --- |
|  | **n=574** | **n=436** | **n=48** | **n=90** |  |
| 2012 | 7 (1.2%) | 4(0.9%) | 2(4.2%) | 1(1.1%) | 0.15 |
| 2013 | 26(4.5%) | 18(4.1%) | 2(4.2%) | 6(6.7%) | 0.57 |
| 2015 | 84(14.7%) | 65(14.9%) | 5(10.4%) | 14(15.6%) | 0.68 |
| 2016 | 172(30%) | 131(30.1%) | 15(31.3%) | 26(28.9%) | 0.95 |
| 2017 | 175(30.4%) | 140(32%) | 10(20.8%) | 25(27.8%) | 0.24 |
| 2018 | 110(19.2%) | 78(17.9%) | 14(29.2%) | 18(20%) | 0.17 |
